# Spectrum of Central Nervous System Involvement in Childhood-Onset Sjögren’s Syndrome: A Case-Based Review

**DOI:** 10.31138/mjr.241123.soc

**Published:** 2024-05-21

**Authors:** Furkan Can Yilmaz, Hakan Kisaoglu, Ozge Baba, Mukaddes Kalyoncu

**Affiliations:** 1 Faculty of Medicine, Karadeniz Technical University, Trabzon, Turkey,; 2 Division of Paediatric Rheumatology, Faculty of Medicine, Karadeniz Technical University, Trabzon, Turkey

**Keywords:** central nervous system, children, headache, Sjögren’s syndrome, treatment

## Abstract

Sjögren’s syndrome (SS) is less frequently diagnosed in childhood than in adults, and central nervous system (CNS) disease is among the rarest systemic involvements. Thus, the clinical spectrum of CNS diseases and their management strategies have not been fully defined. In this article, we present the case of a 16-year-old girl who was referred for severe headache and diagnosed with SS with CNS involvement. Several immunosuppressive treatments failed to improve her neurological symptoms until the rituximab treatment. When we systematically reviewed the literature on cases of CNS involvement in childhood-onset SS, we found that CNS involvement was the presenting feature at the diagnosis of SS in the majority of published cases. While headache and fever were the most frequent complaints at presentation, most of the children displayed features of neuromyelitis optica spectrum disorder. CNS disease showed a variable response to immunosuppressives, and residual neurological deficits were not rare. Additionally, a significant number of cases required treatment with rituximab due to the treatment failures or subsequent flares. Sjögren’s syndrome should be considered in children presenting with predominant neurological symptoms, and careful evaluation of glandular features might help in the prompt diagnosis of childhood-onset SS in children with CNS disease.

## INTRODUCTION

Sjögren’s syndrome (SS) is a chronic systemic autoimmune disease that mainly affects the exocrine glands with lymphocytic infiltration and B-cell hyperactivity.^1^ Involvement in other organ/systems such as the kidney, liver, lung, endocrine glands, nervous system, muscle, joint, or inflammation of the vessel wall might be seen in association with the disease, which is referred to as extraglandular involvement. Central, peripheral, and/or autonomous nervous system involvement can be seen in association with SS, and peripheral nervous system involvement is more common than central nervous system involvement.^2^ Neurological manifestations of SS account for 20% of all SS cases, and central nervous system (CNS) involvement is seen in 1–5% of cases, most commonly as neuromyelitis optica spectrum disorder (NMOSD).^3^ Childhood-onset SS accounts for 5% of all cases, and CNS involvement and NMOSD have been reported in 2% of children with SS.^4,5^ Thus, the spectrum and features of CNS diseases in children with SS are not well-defined. Here, we present a case of SS-related CNS involvement and review the literature to identify the spectrum of CNS disease, treatments, and outcomes in childhood-onset SS.

## CASE REPORT

A 16-year-old female patient was referred to us for the evaluation of autoimmune diseases due to a severe headache. Complaints of headache started a year ago, gradually worsened, and impaired her sleep. Additionally, she reported fatigue, joint pain with morning stifness in the hands, Raynaud’s phenomenon, and dry mouth and eyes. The patient was of Afghan origin and had no known rheumatic disease in her family. Physical examination, including detailed neurological assessment, was normal, and no active arthritis was observed. On laboratory examination, the complete blood count was normal without any cytopenia, and acute phase responses were within normal limits. The patient’s immunoglobulin M (IgM) level was high (2.5 g/L) and other immunoglobulin subclasses were normal. In addition, a low complement C4 (0.08 g/L) was observed with a normal complement C3 level. The rheumatoid factor level was within the normal range, and cryoglobulin levels were not studied. Anti-nuclear antibody was positive with a titer of > 1/1000, but no specific autoantibody was detected in the extractable nuclear antigen panel, including anti-dsDNA, anti-Sm, anti-SSA, anti-SSB, and anti-centromere antibodies. Owing to the symptoms of dryness, a Schirmer test was performed, which revealed severe dry eyes (< 5 mm) in both eyes. In addition, heterogeneity was observed in salivary gland ultrasound. Magnetic resonance imaging (MRI) of the brain revealed multiple small hyperintense lesions in the white matter on T2 weighted images which might be compatible with a demyelinating process or vasculopathy. The patient was diagnosed as CNS involvement of Sjögren’s syndrome, and prednisolone (0.5 mg/kg) with azathioprine treatment was initiated. During follow-up, the patient demonstrated vasculitic rashes in the upper extremities, and the CNS symptoms were not responsive to the initial treatment. Cyclophosphamide with pulse methylprednisolone was employed but patient continued to experiance severe headache. Thus, treatment with rituximab was initiated with mycophenolate mofetil as maintenance therapy. Following the administration of rituximab, the patient’s symptoms disappeared, including fatigue, steroid treatment was withdrawn, and serum complement levels returned to normal. Over a one year period after rituximab treatment, the patient did not display any extraglandular disease manifestations.

## SEARCH STRATEGY

**Figure 1.**
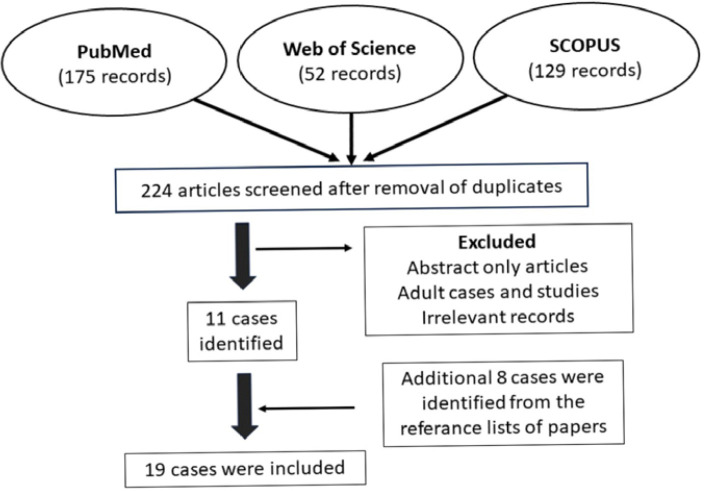
Flow chart of the litreature review.

We aimed to identify case reports or case series of children with childhood-onset Sjögren’s syndrome associated with CNS involvement in the English language. Records were identified from PubMed, Web of Science, and Scopus databases on May 7, 2023. The terms used were “Sjögren” AND “child” AND “nervous system”, and “Sjögren” AND “child” AND “cns”. The initial search revealed 356 records. After the removal of duplicates, titles and abstracts were screened to exclude irrelevant papers, abstract-only publications, and adult cases. Eleven articles remained after the review and additional relevant articles were identified from the reference lists of the papers. After the review, nineteen published cases of childhood-onset CNS disease, associated with SS, were identified (**Figure 1**). Age at onset, sex, glandular and extraglandular features, CNS disease characteristics, laboratory and imaging results, and treatments were retrieved from the publications.

## DISCUSSION

Herein, we describe a case of an adolescent with Sjögren’s syndrome and associated CNS involvement without any overt neurological findings. In addition, our literature review showed that despite almost half of the children displayed NMOSD, CNS disease in childhood-onset SS has a wide spectrum of features, including cranial nerve palsies, seizure disorders, aseptic meningitis, and encephalopathy. In addition, a high rate of variability was observed among the treatment modalities, but rituximab was used in the majority of cases published after 2015. The most frequent symptoms in published cases were headache, fever, and weakness, and SS was frequently diagnosed at the onset of CNS disease. The disease characteristics of the published cases of Sjögren’s syndrome are shown in **Table 1** and **Table 2**.

**Table 1.** Clinical and laboratory features of childhood-onset Sjögren’s syndrome reported in the literature.

**Case no -year**	**Age-gender**	**Glandular features**	**Extra glandular features**	**Immunoglobulins**	**ANA**	**anti-SS-A**	**Complement status**
#1 – 1990^6^	10 - F	dry eyes	Fever	High IgG Normal IgM/A	1:160	Positive	Normal
#2 – 1995^7^	9 - F	dry eyes, submandibular swelling	leukopenia	High IgG	Elevated	Positive	Normal
#3 – 1996^8^	9 - F	dry eyes, parotid swelling	fever, renal tubular acidosis	High IgG Normal IgM/A	1:1280	Positive	Normal
#4 – 1998^9^	14 - M	dry eyes, dry mouth, submandibular swelling	fever, uveitis	High IgG Normal IgM Normal IgA	Negative	Negative	Normal
#5 – 2001^10^	4 - F	dry eyes, dry mouth, parotid swelling	fever, anemia	N/M	1:2560	Positive	Normal
#6 – 2004^11^	16 - F	dry mouth, parotid swelling	N/M	N/M	1:1280	Positive	Normal
#7 – 2006^12^	11 - F	Sicca symptoms	N/M	N/M	1:160	Positive	N/M
#8 – 2008^13^	16 - F	parotid swelling	fever, thrombocytopenia	High IgG High IgM Normal IgA	Elevated	Positive	Normal C3
#9 – 2008^14^	12- F	N/M	N/M	High IgG	1:640	Positive	Normal C3
#10 – 2016^15^	6 - F	None	fever	Normal	1:160	Negative	Normal
#11 – 2016^16^	9 - F	recurrent keratoconjuctivitis, dry mouth	Fever, renal tubular acidosis	High IgM High IgG	1:80	Positive	N/M
#12 – 2017^17^	13 - F	dry eye and mouth	N/M	N/M	Elevated	Positive	N/M
#13 – 2017^18^	14 - M	dry mouth	N/M	N/M	N/M	Positive	N/M
#14 – 2019^19^	4 - M	dry mouth, submandibular and parotid swelling	N/M	N/M	N/M	Positive	N/M
#15 – 2020^20^	12 - F	dry eyes, dry mouth	Fever, glucosuria	Normal IgG/A/M	1:100	Negative	Normal
#16 – 2021^21^	15 - F	None	thrombocytopenia	Normal IgG	Elevated	Positive	Normal
#17 – 2022^22^	18 - F	N/M	fever	High IgG	1:2560	Positive	Normal
#18 – 2022^23^	16 - F	dry eyes	interstitial lung disease	N/M	N/M	Positive	N/M
#19 – 2022^24^	5 - F	parotid swelling	fever, interstitial lung disease, liver disease	High IgG	Elevetad	Positive	Low C3 Low C4
#20 – current case	16 - F	dry eyes, dry mouth	arthritis, Raynaud’s phenomenon	High IgM Normal IgG/A	>1:1000	Negative	Normal C3 Low C4

ANA: anti-nuclear antibody; SSA: Sjögren’s syndrome related protein A; F: female; M: male; Ig: immunoglobulin; C3: complement C3; C4: complement C4; N/M: not mentioned.

**Table 2.** Features of central nervous system involvement, treatments, and outcomes of published cases with childhood-onset Sjögren’s syndrome.

**Case number**	**Presentation**	**Diagnosis of SS**	**Neurological manifestations**	**MRI**	**Treatments employed**	**Neurological outcome**
#1 – 1990^6^	weakness, headache, dizziness, blurry vision	At the onset of CNS disease	loss of proprioception and touch sensation of right arm	multiple infarcts at pons, midbrain, bilaterally in the temporal lobes and cerebellar hemispheres	GC, CsA	Decreased visual acuity
#2 – 1995^7^	headache, weakness	At the onset of CNS disease	hemiparesis, meningeal irritation, anisocoria	multiple T2-weighted hyperintensities at white matter and from C1 to T8, swelling of spinal cord	GC	Mild residual paresis with subsquescent flare
#3 – 1996^8^	headache	At the onset of CNS disease	meningeal irritation	T2-weighted hypo intensity at right deep temporal lobe	GC, CYC	Complete recovery
#4 – 1998^9^	seizure, weakness, acute loss of vision and hearing	At the onset of CNS disease	meningoencephalitis, sensorineural hearing loss, central facial paresis, bowel/bladder incontinence, spasticity, hyperreflexia	multiple T2-weighted hyperintensities at white matter and thoracolumbar spinal cord, diffusely enlarged right fifth cranial nerve, cerebral atrophy	GC, CYC	Partial vision loss, difficulty walking, lost bladder control
#5 – 2001^10^	headache, dizziness, ataxia	N/M	vertical diplopia, left oculomotor palsy, right superior oblique palsy	Tl-weighted low signal intensity in the dorsal midbrain focal contrast enhancement in the left dorsal midbrain	GC	Facial diplegia
#6 – 2004^11^	headache	Prior to the CNS disease	ataxia, ocular dysmetria, cerebellar dysarthria	T2-weighted hyperintense lesions at cerebellum with mild contrast enhancement	GC	Recovery
#7 – 2006^12^	weakness	At the onset of CNS disease	paraparesis, urinary incontinence, sensory deficit	optic neuritis and T2-weighted hyperintense lesions from C1 to the conus	GC, PLX, CYC, RTX	Partial vision loss
#8 – 2008^13^	headache, weakness	At the onset of CNS disease	sensory disturbance, muscular weakness	T2-weighted hyperintensity at lateral ventricle and longitudinal lesion from C5 to T10	GC, TAC	Recovery
#9 – 2008^14^	difficulty using right hand, jerky movements	At the onset of CNS disease	right sided ataxia	left cerebellar atrophy	GC, IVIG, MTX	N/M
#10 – 2016^15^	headache, unilateral weakness, altered mental status	At the onset of CNS disease	progressive right sided motor weakness, mild abducens and facial weakness	multiple T2-weighted hyperintensities at white matter	GC, RTX, AZA	Mild weakness
#11 – 2016 ^16^	seizure, altered mental status	At the onset of CNS disease	automonic dysfunction, urinary incontinance, upper gaze deviation	Diffuse hyperintensity in cerebellar hemispheres	GC, IVIG	Residual gaze deviation and ataxia
#12 – 2017^17^	blurry vision, headache	At the onset of CNS disease	Gaze deviation	Hyperintense lesions in internal capsule and T2 patchy lesions in thoracic and cervical spine	GC, HCQ, PLX, CYC, MMF, RTX	N/M
#13 – 2017 ^18^	vision loss, unilateral weakness	At the onset of CNS disease	Left sided vision loss, right hemiparesis	Left-sided white matter lesion and T2 hyperintense lesions through T2-T5	GC, PLX, CYC, MMF, RTX, TOC	N/M
#14 – 2019^19^	seizure	At the onset of CNS disease	generalised tonic clonic convulsion	Normal	CsA, GC	Seizure disorder
#15 – 2020^20^	headache	Prior to the CNS disease	meningeal irritation, sensory deficit in upper extremities	Normal	NSAII, HCQ, SLZ, GC	Complete recovery
#16 – 2021^21^	headache, blurry vision	At the onset of CNS disease	diplopia, decreased movement of left eye	Normal	HCQ, GC, RTX	Complete recovery
#17 – 2022^22^	headache	N/M	none	N/M	GC, MTX	Complete recovery
#18 – 2022^23^	headache, dizziness	At the onset of CNS disease	None	T2 weighted-hyperintense lesions with gadolinium enhancement in the brain, brainstem, and dorsal spine	GC, HCQ, MMF, RTX	N/M
#19 – 2022^24^	decline in vision	Prior to the CNS disease	None	left optic atrophy and swelling of the spinal cord at the levels of c2-6	GC, MMF, TAC, HCQ, RTX, IVIG	Partial vision loss
#20 – current case	headache	At the onset of CNS disease	None	T2 – weighted hyperintense lesions at white matter	GC, AZA, MMF, CYC, RTX	Complete recovery

CNS: central nervous system; SS: Sjögren’s syndrome; MRI: magnetic resonance imaging; NM: not mentioned; GC: glucocorticoid; TAC: tacrolimus; HCQ: hydroxychloroquine; RTX: rituximab; AZA: azathioprine; MTX: methotrexate; PLX: plasmapheresis; TOC: tocilizumab; CsA: cyclosporine; SLZ: sulphasalasine; CYC: cyclophosphamide; MMF: mycophenolate mofetil; IVIG: intravenous immunoglobulin

Childhood-onset Sjögren’s syndrome can occur at any age and accounts 1–5% of cases.^3,25^ Unlike in adults, sicca symptoms were less frequently detected in children, and recurrent parotiditis might be the only presenting sign of glandular disease. Thus, in some children, diagnosis requires imaging and histopathological evaluation of glandular involvement.^26^ The most recent ACR/EULAR classification criteria for Sjögren’s syndrome do not cover the full spectrum of childhood-onset disease, and children are less frequently evaluated for the features of the classification criteria.^25^ Similarly, our patient do not fulfill the classification criteria, but a glandular biopsy was thought to be unnecessary to conclude a diagnosis due to the classical picture of the disease in our case. Likewise, the majority of published cases of CNS disease displayed sicca symptoms. Thus, rigorous assessment of glandular features may improve the diagnosis of childhood-onset SS. Additionally, the majority of published cases display a positive anti-SSA antibody. Since, autoantibodies are evident before the development of symptoms in adults with SS,^27^ it might be speculated that the features of SS might be evident before the onset of CNS disease in children.

The association between NMOSD and SS is well known in adult-onset disease.^2,3^ Similarly, our study showed that almost half of the children with CNS involvement displayed the features of NMOSD. In addition, variable presentations such as aseptic meningitis, meningoencephalitis, and seizure disorders might be observed. Despite not being included in this systematic review, psychiatric manifestations associated with childhood-onset SS have been also reported.^28^ Studies have shown that low complement C3, male sex, anti-SSA, and kidney and pulmonary involvement might be associated with neurological involvement in adults with SS.^29,30^ However, in published cases of childhood-onset SS, fever was the most common extraglandular feature, and other involvements such as kidney or lung involvement were rarely reported. In addition, hypocomplementemia was not frequently observed.

Magnetic resonance imaging (MRI) has no place in the current diagnostic criteria for neurological manifestations of SS, but it is useful for narrowing the differential diagnosis. The most common presentation of Sjögren’s syndrome on MRI, regardless of patient age, is periventricular or subcortical, focal and nonspecific, T2-hyperintense lesions.^31^ Similarly, the majority of published cases of chilhood-onset SS with CNS involvement displayed changes in intensity or abnormalities on T2-weighted MRI.

Treatment of SS can be divided into two categories: relief of the main symptoms and treatment of severe systemic disease, and treatment should be personalised on a case basis.^32,33^ A recent systematic review showed that rituximab is most commonly used for the treatment of kidney and CNS diseases in childhood-onset SS.^34^ Similarly, our study showed that rituximab was frequently employed in chilhood-onset SS with CNS involvement; however, these patients were also treated with a varying range of immunosuppressives with either treatment failures or flares, which resulted in the use of rituximab. The latest EULAR recommendations for the treatment of SS also state that systemic immunosuppressants should be used only in the presence of systemic disease. In addition, for the management of CNS disease, cyclophosphamide was favored over rituximab as the first-line treatment, especially for NMOSD and vasculitic presentations.^35^

Despite the higher prevalence of SS in adults, the prevalence and association of residual neurological deficits have not been investigated in SS patients with neurological involvement. However, our study showed that residual neurological deficits are not uncommon in childhood-onset SS with CNS involvement. Interestingly, among the three cases with CNS involvement during the course of SS, only one required aggressive immunosuppression and developed neurological sequelae. In addition, complete neurological recovery is frequently reported in children receiving rituximab treatment. Thus, it can be speculated that early recognition of glandular disease, before the onset of CNS involvement might improve patient outcomes.

In conclusion, while almost half of CNS involvement in childhood-onset SS shows features of neuromyelitis optica spectrum disorder, CNS disease displays a high degree of variability, such as aseptic meningitis and encephalopathy. In most children, diagnosis of childhood-onset SS is at the time of the CNS involvement. Thus, prompt diagnosis requires careful evaluation of glandular and serological features. The treatment response does not seem to be associated with the presence of prominent neurological symptoms, but it is important to keep in mind that CNS disease might result in a residual neurological deficits. A wide variety of agents are used for treatment, but they result in different success rates on a case basis. However, most children require treatment with rituximab, which seems to be effective in the treatment of CNS involvement.
